# Collective migration during a gap closure in a two-dimensional haptotactic model

**DOI:** 10.1038/s41598-021-84998-w

**Published:** 2021-03-12

**Authors:** Marie Versaevel, Laura Alaimo, Valentine Seveau, Marine Luciano, Danahe Mohammed, Céline Bruyère, Eléonore Vercruysse, Olivier Théodoly, Sylvain Gabriele

**Affiliations:** 1 Mechanobiology & Soft Matter Group, Interfaces and Complex Fluids Laboratory, Research Institute for Biosciences, CIRMAP, University of Mons, 20 Place du Parc, 7000 Mons, Belgium; 2 Adhesion and Inflammation Laboratory, INSERM U1067, UMR 7333, CNRS, 163 avenue de Luminy-Case 937, 13288 Marseille Cedex 09, France

**Keywords:** Cellular motility, Biomedical engineering

## Abstract

The ability of cells to respond to substrate-bound protein gradients is crucial for many physiological processes, such as immune response, neurogenesis and cancer cell migration. However, the difficulty to produce well-controlled protein gradients has long been a limitation to our understanding of collective cell migration in response to haptotaxis. Here we use a photopatterning technique to create circular, square and linear fibronectin (FN) gradients on two-dimensional (2D) culture substrates. We observed that epithelial cells spread preferentially on zones of higher FN density, creating rounded or elongated gaps within epithelial tissues over circular or linear FN gradients, respectively. Using time-lapse experiments, we demonstrated that the gap closure mechanism in a 2D haptotaxis model requires a significant increase of the leader cell area. In addition, we found that gap closures are slower on decreasing FN densities than on homogenous FN-coated substrate and that fresh closed gaps are characterized by a lower cell density. Interestingly, our results showed that cell proliferation increases in the closed gap region after maturation to restore the cell density, but that cell–cell adhesive junctions remain weaker in scarred epithelial zones. Taken together, our findings provide a better understanding of the wound healing process over protein gradients, which are reminiscent of haptotaxis.

## Introduction

Despite the role of gradients of proteins in physiological^1,2^ and pathological^3,4^ situations, most of the in vitro studies in cellular biology are conducted on cells grown on bidimensional culture substrates which are coated homogeneously with adhesive proteins^5^. It has been reported that normal and cancer cell motility can be directed by a protein-bound gradient^6,7^, whereas neurogenesis^8,9^ and immune response^10,11^ also rely on the cellular response to a varying concentration of bound-proteins. The directional migration of cells in response to gradients of substrate-bound proteins is termed haptotaxis and its understanding requires the development of bioengineering techniques to design well-controlled gradients of proteins on culture substrates^12^. During the past decades, few methods have emerged to create protein gradients such as microfluidics^8,13,14^, photochemistry^15,16^ and microcontact printing^17–19^, but most of these techniques are time-consuming and difficult to carry out, especially for making large zones of protein gradients.

Here we created well-defined gradients of fibronectin over distance of hundreds of microns by using the maskless and contactless photolithography PRIMO method^20,21^. We grew Martin-Darby Canin Kidney (MDCK) epithelial cells^22^ on flat culture substrates covered with circular gradients of fibronectin (FN). MDCK cells preferentially adhere and spread on the regions with a high density of adhesive proteins, forming rounded gaps over circular FN gradients that enable to study the mechanisms of gap closure in haptotactic conditions. Epithelial tissues close open gaps slower on FN gradients than on homogeneous FN coatings by increasing significantly the spreading areas of leader cells. This mechanism allows to close open gaps regardless the gap geometry and leads to a lower cell density in freshly closed gap regions, which is restored after 36 h by increasing the proliferation rate. In addition, we found a weakening of cell–cell adhesive junctions in gaps closed over a FN gradient.

## Results

### Gap closure dynamics was slowed down on FN gradient compared to homogeneous FN coatings

**Figure 1:**
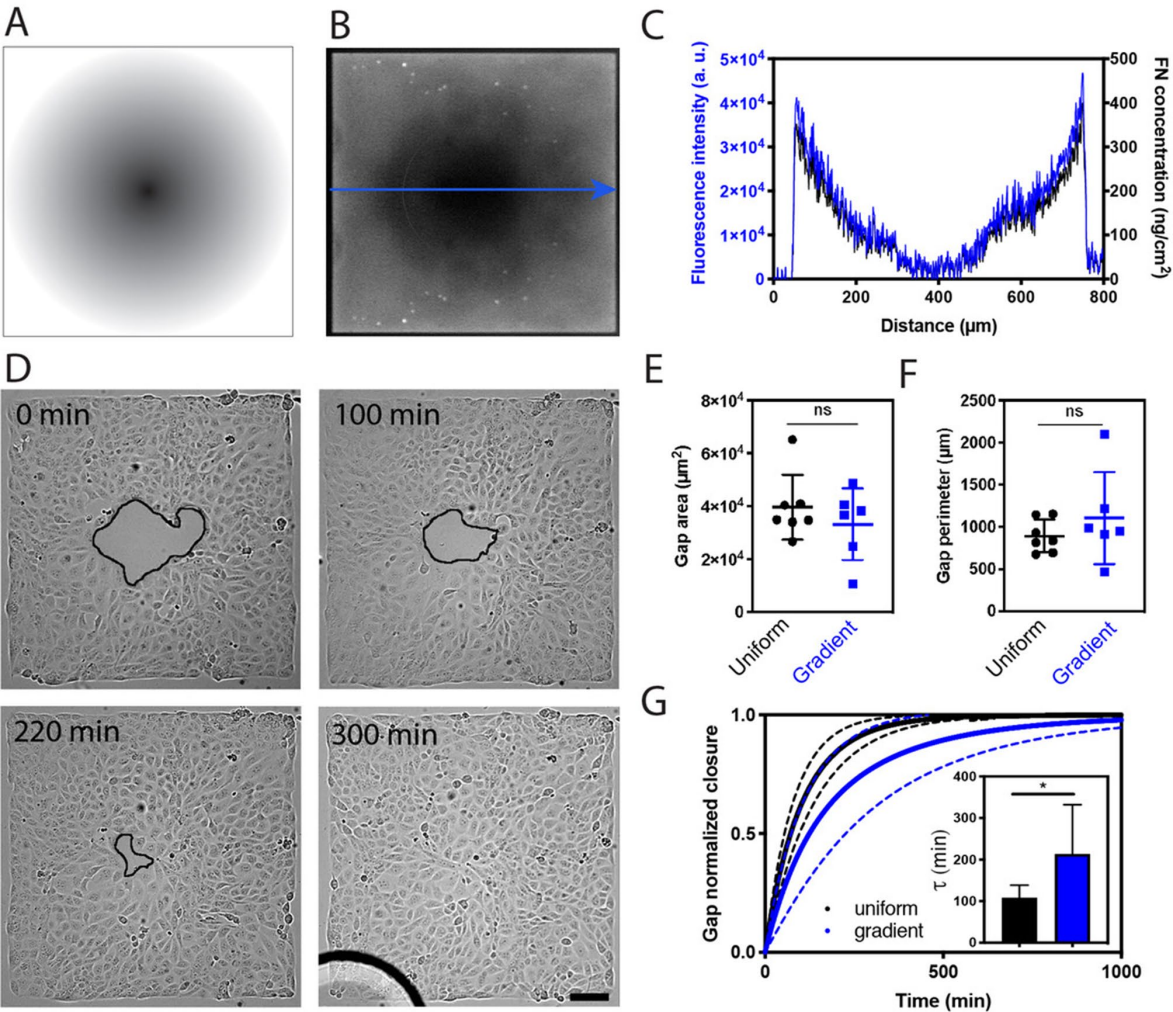
Epithelial gaps closure is slowed down on a FN gradient compared to on homogeneously coated substrates. (**A**) Design of the photopattern used to create a centered circular gradient of FN (gradient of gray) within a square FN pattern (in white) of 764 µm wide. (**B**) Epifluorescent image of a FN gradient labelled with rhodamine. (**C**) Plot profile of the fluorescence intensity (left axis) and the surface density (right axis) of the rhodamine-conjugated FN according to the blue arrow. Low fluorescence intensity values at both extremities correspond to the black frame of (**B**). (**D**) Time-lapse images in DIC mode of a gap closure in a confluent epithelium grown on a circular FN gradient. The edge of the closing gap was highlighted with a black line. Scale bar, 100 µm. Distribution of (**E**) the gap area and (**F**) the gap perimeter in confluent epithelia grown on a uniform coating (in black) or a gradient (in blue) of FN. (**G**) Temporal evolution of the mean normalized gap area during the closure on a uniform coating (in black, n = 7) or a gradient (in blue, n = 6) of FN. Full lines represent the mean and dashed lines the S.D.

We studied whether haptotaxis can modulate the dynamics of gap closure in bidimensional epithelia by using a photopatterning technique (PRIMO, Alvéole) to create square patterns of 764 µm × 764 µm with a radial gradient of fibronectin (FN) of 764 µm in diameter (Fig. 1 A and B). The concentration of FN decreased towards the center of the radial pattern, as indicated by the plot profile of the fluorescence intensity of rhodamine-labelled FN (Fig. 1C), covering a FN density ranging from 384 ± 10 ng/cm^2^ for the zone located at the periphery to 32 ± 6 ng/cm^2^ for zone located at the center of the pattern (Supplementary Figure S1)^23^. As a consequence, we defined a zone of high FN density at the periphery of the pattern and a circular zone of low FN density that formed a gradient towards the center of the pattern. MDCK cells were seeded at 80,000 cells/cm^2^ on square patterns with a radial FN gradient, corresponding to the formation of minimal epithelial sheets of ~ 500 cells distributed on a square area of 0.583 mm^2^. As shown in Fig. 1D, MDCK epithelial cells attached and spread preferentially at the periphery of the pattern, corresponding to the high FN density. At short experimental times, we observed the formation of a circular gap centered on the region of lower FN density (Fig. 1D), corresponding to 7 ± 2% (n = 6) of the total tissue area of the square pattern.

We used time-lapse microscopy for studying the closure dynamics of epithelial gaps on radial FN gradients (Fig. 1D, Supplementary Figure S2, Movie S1–S3). We selected circular gaps of 33,000 ± 14,000 µm^2^ (Fig. 1E) with a mean perimeter of 825 ± 350 µm (Fig. 1F) to ensure that we compared similar gap geometries on gradient and control uniform FN coatings, for which gaps were created with PDMS stencils^24,25^ of 200 µm in diameter (Supplementary Figure S3 and Supplementary Movie S2 and S4). Then we plotted the temporal evolution of the normalized gap area during the closure on gradient and uniform FN coatings. As shown in Fig. 1G, we found that the gap closure was slightly delayed on FN gradients, with a characteristic time τ = 214 ± 118 min, compared to τ = 106 ± 28 min on a homogeneous FN coating with a high FN density around 1 µg/cm^2^. Our results indicated therefore that decreasing the FN density with gradient pattern leads to a slowdown of the epithelial gap closure.

### Gap closures on FN gradients require a larger spreading of leader cells

**Figure 2:**
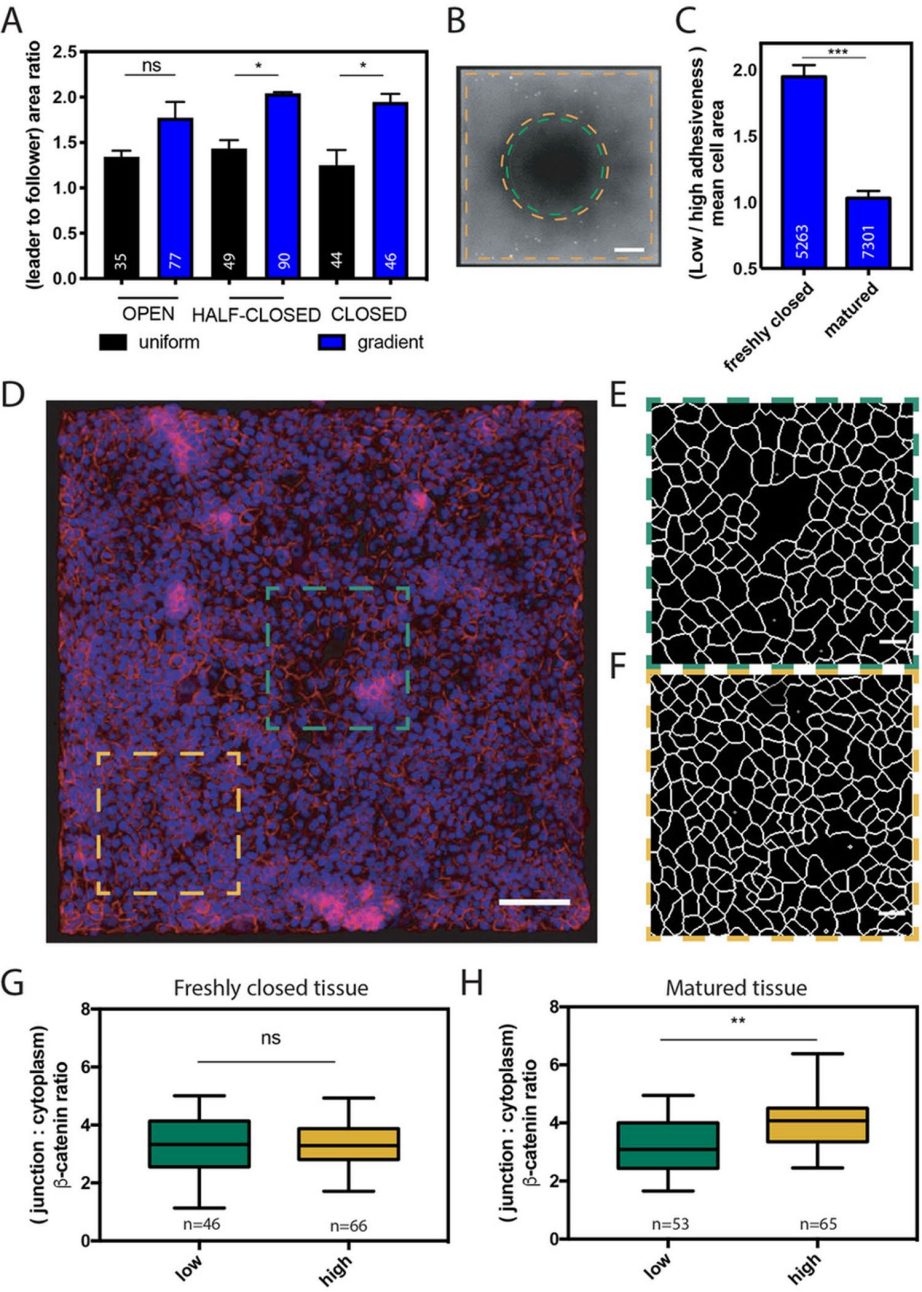
Gap closure on a gradient of FN requires more cell spreading and leads to weaker cell–cell contacts. (**A**) Ratio of the cell area between leader and follower cells during a gap closure on a uniform FN coating (density ~ 1 µg/cm^2^ in black) and a FN gradient (in blue). (**B**) The FN pattern was divided in two zones: high FN density zone (from 384 ± 10 down to 118 ± 8 ng/cm^2^) at the periphery (in orange) and a low FN density zone (from 118 ± 8 down to 32 ± 6 ng/cm^2^) at the center (in green). (**C**) Ratio of the cell areas on low FN density zone (from 118 ± 8 down to 32 ± 6 ng/cm^2^) versus high FN density zone (from 384 ± 10 down to 118 ± 8 ng/cm^2^). Cell areas were measured at t = 3 h (freshly closed tissue, n = 3) and t = 36 h (matured tissue, n = 4) after gap closure. (**D**) Fluorescent image of a gap freshly closed over a FN gradient. Nuclei were labelled in blue with DAPI and β-catenin was labelled in red. The scale bar is 100 µm. Segmentation of the cell–cell junctions in epithelial tissues over (**E**) low (FN density from 118 ± 8 down to 32 ± 6 ng/cm^2^) and (**F**) high (FN density from 384 ± 10 down to 118 ± 8 ng/cm^2^) FN zones. The scale bars are 20 µm. (**G** and **H**) Ratio of the fluorescence intensity of β-catenin between cell–cell junctions and cytoplasmic zones of cells grown on low (FN density from 118 ± 8 down to 32 ± 6 ng/cm^2^, in green, n = 2) and high (FN density from 384 ± 10 down to 118 ± 8 ng/cm^2^, in orange, n = 2) FN zones for epithelial monolayers (**G**) freshly closed or (**H**) matured over a FN gradient.

Previous works have shown that epithelial wound healing depends on the migration of multicellular assemblies, which are pulled by leader cells localized at the gap edge^24,26,27^. Differences in spatial confinement between followers and leader cells in growing epithelial tissues have been shown to modulate their cell migration velocities^28^. Leader cells located at the front of the tissue are larger and faster than follower cells, which are densely packed at the rear (Supplementary Figure S2). Based on this observation, we assumed that the spreading area of epithelial cells may be modulated by the FN ligand density during wound healing events. As shown on Supplementary Figure S4, we measured the spreading area of follower and leader cells at three main stages of the gap closure: open (i.e. 100% of the initial gap area), half-closed (i.e. 50% of the initial gap area) and closure (i.e. 0% of the initial gap area) on uniform coatings and radial FN gradients (Fig. 2A). Our findings revealed that leader cells on radial FN gradients exhibited larger spreading areas than followers, regardless the stage of the closure process. As shown on Fig. 2A, the ratio of spreading areas between leaders and followers was systematically larger on FN gradients (1.95 ± 0.09) than on homogenous FN coatings (1.25 ± 0.17) at the closure time. We next investigated whether the spreading difference between leaders and followers was conserved in matured tissues. To answer this question, we first defined a circular region of interest (ROI) of 350 µm in diameter centered with the square pattern and corresponding to a zone of low cell-ligand density (FN density from 118 ± 8 down to 32 ± 6 ng/cm^2^, Fig. 2B, in green). The rest of the pattern minus a border stripe of 10 µm to exclude border cells was used to create a second ROI, corresponding to a zone of high cell-ligand density (with FN density from 384 ± 10 ng/cm^2^ down to 118 ± 8 ng/cm^2^ Fig. 2B, in orange). β-Catenin-stained images of MDCK freshly closed tissues or matured tissues at 36 h after closure were acquired by epifluorescence microscopy and then segmented with EpiTools software^29^ to detect cell–cell junctions^30^ and quantify individual cellular areas in both ROIs. Interestingly, the ratio of spreading areas between leaders and followers, which was 1.95 ± 0.09 for freshly closed gaps on FN gradients, dropped to 1.03 ± 0.05 after 36 h of maturation (Fig. 2C).

Altogether, these findings indicate that gap closures on FN gradients require larger spreading of leader cells, whereas the significant difference of cellular areas between zones of low (FN density from 118 ± 8 down to 32 ± 6 ng/cm^2^, Fig. 2E) and high (FN density from 384 ± 10 down to 118 ± 8 ng/cm^2^, Fig. 2F) cell-ligand density in freshly closed gaps was restored after 36 h of tissue maturation, leading to a constant cellular density.

### Epithelial wound healing over a FN gradient leads to weaker cell–cell junctions

Adhesion events implicated in the maintenance of the mechanical integrity of epithelial tissues are mediated by cadherin and integrin adhesion receptors^31,32^. Increasing evidence of a dynamic crosstalk between both adhesion complexes through common signaling pathways suggest that a modulation of cell-substrate ligand density may affect cell–cell junctions^33^. Cell–cell adhesive interactions have been shown to maintain the cohesion of dense epithelial tissues and are also implicated in the modulation of the collective motion in epithelia^34^. We examined whether a gap closure over a FN gradient could modulate cell–cell adhesive interactions by quantifying the contrast of β-catenin between cell–cell junctions and the cytoplasm (Fig. 2D and Supplementary Figure S5). The junction to cytoplasm β-catenin ratio was measured in freshly closed (t = 3 h after closure, Fig. 2G) and mature (t = 36 h after closure, Fig. 2H) tissues for zones of low (FN density from 118 ± 8 down to 32 ± 6 ng/cm^2^, Fig. 2E) and high (FN density from 384 ± 10 down to 118 ± 8 ng/cm^2^, Fig. 2F) cell-ligand density. As shown in Fig. 2G, we observed similar β-catenin fluorescence intensity ratios on low and high cell-ligand density areas in freshly closed tissues. Interestingly, our findings indicated that the β-catenin ratio in matured tissues was increased in zones of high cell-ligand density, whereas it remained constant in zones of low cell-ligand density (Fig. 2H). Our findings indicate therefore that cell–cell adhesive junctions in mature epithelia are weaker in a gap region closed over a FN gradient.

### Proliferation restores the balance of cell density after closure

**Figure 3:**
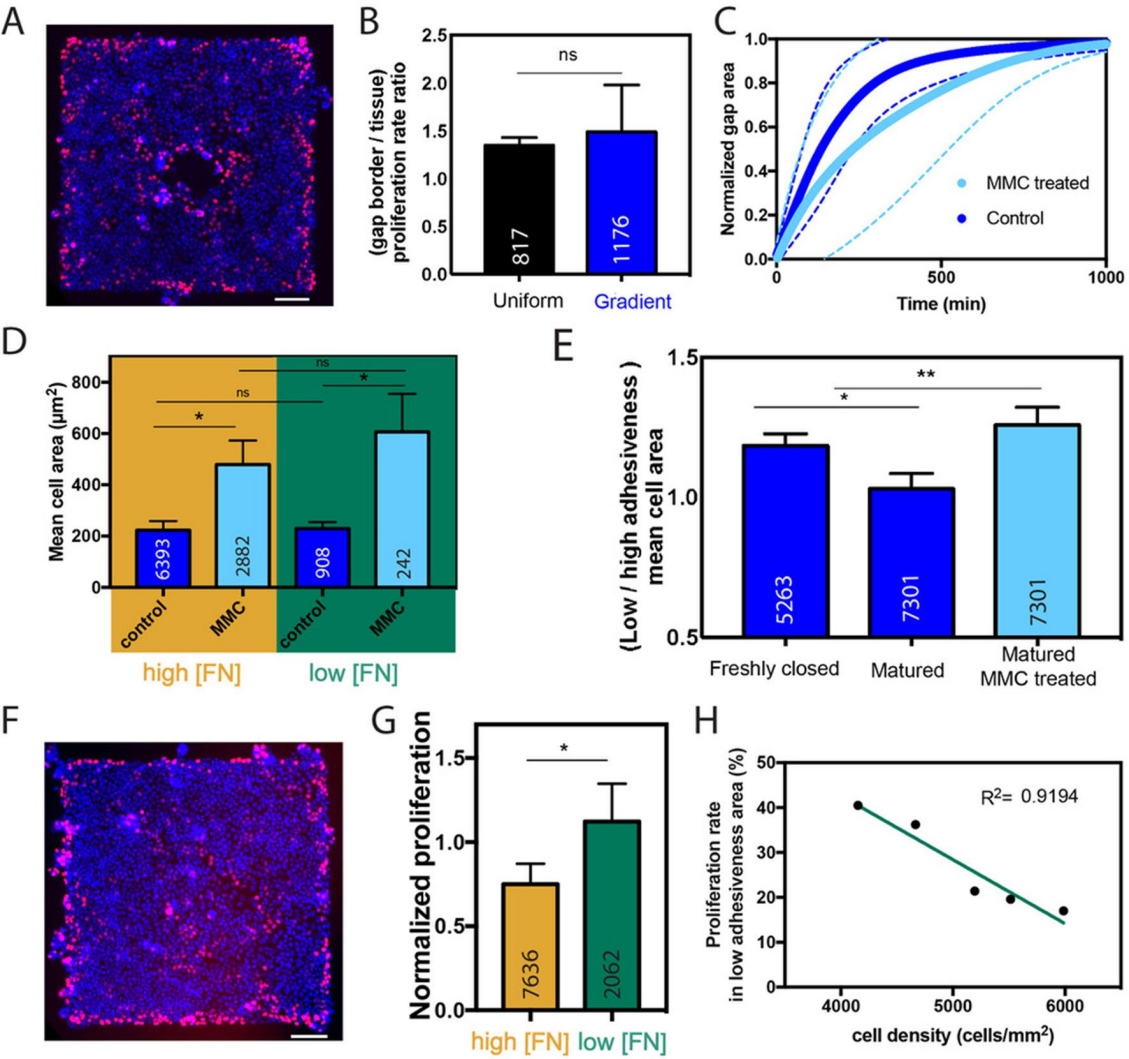
Cell proliferation increased on the low fibronectin density region of the gradient to rebalance the cell density. (**A**) Edu-labelling of proliferating cells (in red) during an epithelial gap closure over a radial FN gradient. Nuclei were labelled in blue with DAPI. The scale bar is 100 µm. (**B**) Proliferation ratio of leader versus follower cells within epithelial tissues grown on a uniform FN coating (in black) and radial FN gradient (in blue). Data were obtained from 4 individual experiments for each condition. (**C**) Temporal evolution of the normalized gap closure area on a FN gradient for control cells (dark blue, n = 6) and mitomycin-C treated cells (light blue, n = 6). (**D**) Ratio of the cell area over low (FN density from 118 ± 8 down to 32 ± 6 ng/cm^2^, in green) and high (FN density from 384 ± 10 down to 118 ± 8 ng/cm^2^, in orange) FN zones of a radial gradient, for control cells (in dark blue) and mitomycin-C treated cells (in light blue). Data were obtained from 3 individual experiments for each condition. (**E**) ratio of the cell area over low (FN density from 118 ± 8 down to 32 ± 6 ng/cm^2^), and high (FN density from 384 ± 10 down to 118 ± 8 ng/cm^2^) FN zones of a radial FN gradient few hours after the gap closure (freshly closed, dark blue), 32 h after closure (matured, dark blue) and for mitomycin-C treated cells (light blue). Data were obtained from 3 individual experiments for each condition. (**F**) Edu-labelling of proliferating cells (in red) just after the gap closure on a radial FN gradient. Nuclei were labelled in blue with DAPI. The scale bar represents 100 µm. (**G**) Normalized percentage of proliferating cells on high (FN density from 384 ± 10 down to 118 ± 8 ng/cm^2^, in orange) and low (FN density from 118 ± 8 down to 32 ± 6 ng/cm^2^) FN zones of a radial FN gradient. The number at the bottom of the bars correspond to the number of cells. Data were obtained from 5 individual experiments for each condition. (**H**) Proliferation in an epithelial monolayer freshly closed over the low FN zone (FN density from 118 ± 8 down to 32 ± 6 ng/cm^2^) of a gradient versus the cellular density of the monolayer. The green line corresponds to a linear fit (R^2^ = 0.9194).

Even though our findings suggest a modulation of cell spreading area through weak intercellular adhesions during epithelial gap closure on FN gradients, we could not exclude a possible role of cell proliferation. Epithelial tissues grown on uniform FN coatings and FN gradients were treated with the thymidine analogue 5-Ethynyl-2´-deoxyuridine^35^ (EdU) for 45 min in order to identify proliferating cells. As shown in Fig. 3A, proliferating cells were concentrated at the periphery of the pattern and at the border of the closing gap. Previous works have suggested that zones of high cell proliferation in epithelia correspond to regions of higher mechanical stress^36,37^. Interestingly, we showed that the ratio of proliferation rate between leader and follower cells was statistically similar on uniform FN coatings with FN density around 1 µg/cm^2^ and FN gradients with FN density ranging from 384 ± 10 ng/cm^2^ to 32 ± 6 ng/cm^2^ (Fig. 3B). Then we studied the role of cell proliferation on gap closure dynamics by measuring the gap area over time on FN gradients for MDCK tissues treated with mitomycin C (MMC), a DNA-alkylating agent that inhibits cell proliferation^38,39^ . MDCK cells were treated with MMC after forming a circular open gap in a cohesive tissue grown over a FN gradient. Our results showed that MMC-treated tissues closed epithelial gaps with a similar dynamics than control tissues (Fig. 3C), suggesting that cell proliferation was not a key player of gap closure, in agreement with previous observations on homogeneous FN coated surfaces^40^. Surprisingly, our findings indicated that MDCK cells were more than two times larger in MMC-treated tissues than in control ones (Fig. 3D). Indeed, matured tissues treated with MMC and closed over a FN gradient presented larger cells in the zone of low FN density from 118 ± 8 down to 32 ± 6 ng/cm^2^ and smaller cells in the zone of high FN density with FN density from 384 ± 10 down to 118 ± 8 ng/cm^2^ (Fig. 3E). These results suggest that even if cell proliferation does not drive gap closure, its inhibition leads to an imbalance of cell areas that persists in matured tissues. To gain more insight into the mechanism that allows to restore the cell density in scarred tissues, we next consider the role of cell proliferation after closure in EdU-stained tissues (Fig. 3F). We observed a higher proliferation rate in the zone of low FN concentration (from 118 ± 8 down to 32 ± 6 ng/cm^2^) few hours after the gap closure over a FN gradient (Fig. 3G). Interestingly, we found that the proliferation rate in the zone of low FN density (from 118 ± 8 down to 32 ± 6 ng/cm^2^) was linearly related to the cell density (R^2^ = 0.9194, n = 5), suggesting that cell proliferation increases in the closed gap region to restore the balance of cell densities.

### The closure mechanism is not mediated by the geometry of the FN gradient

**Figure 4:**
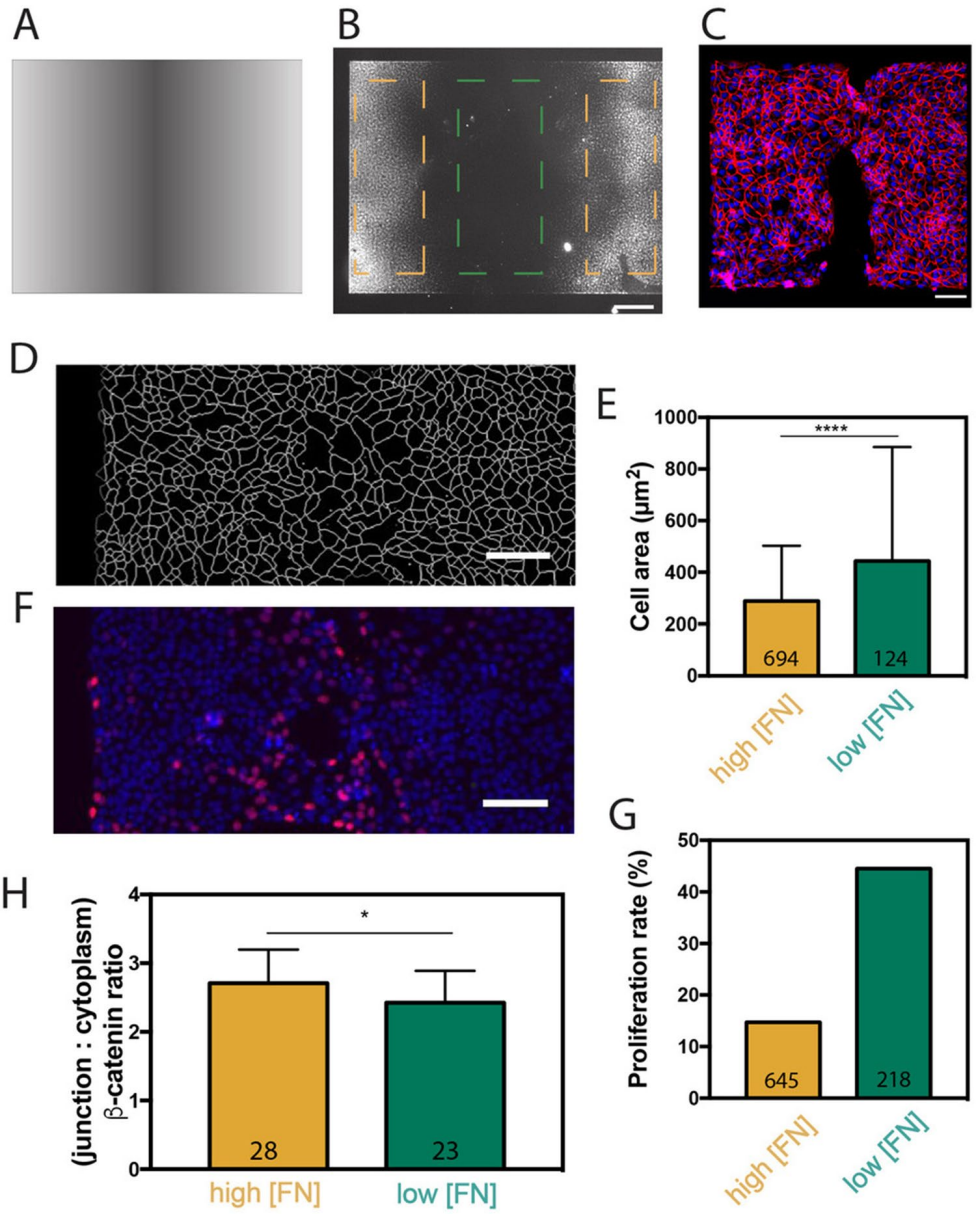
The closure of an epithelial tissue in haptotactic conditions does not depend on the geometry of the gradient. (**A**) Photopattern used to create a linear fibronectin gradient. (**B**) Zoomed picture of the linear FN gradient labelled with rhodamine. The picture corresponds to the half of the total pattern. The adhesive pattern can be divided into high FN zones (FN density from 384 ± 10 down to 118 ± 8 ng/cm^2^, in orange) and a low FN zone in the center (FN density from 118 ± 8 down to 32 ± 6 ng/cm^2^, in green). The scale bar represents 100 µm. (**C**) MDCK tissue grown for 18 h on a linear FN gradient. Nuclei are labelled in blue and β-catenin in red. Scale bar represents 100 µm. (**D**) Result of the segmentation of a MDCK tissue freshly healed over a FN gradient. The scale bar represents 100 µm. (**E**) Area of cells that form the tissue over the high (FN density from 384 ± 10 down to 118 ± 8 ng/cm^2^, in orange) and low (FN density from 118 ± 8 down to 32 ± 6 ng/cm^2^, in green) regions of the FN gradient. (**F**) Edu-labelling of proliferating cells (in red) just after a gap closure over a FN gradient. Nuclei are labelled in blue with DAPI. The scale bar represents 100 µm. (**G**) Percentage of proliferating cells on low and high FN zones of a linear gradient. (**H**) Ratio of the fluorescence intensity of β-catenin in cell–cell junctions and in the cytoplasm of cells over high (FN density from 384 ± 10 down to 118 ± 8 ng/cm^2^, in orange) and low (FN density from 118 ± 8 down to 32 ± 6 ng/cm^2^, in green) FN zones of a liner gradient.

Previous reports have demonstrated that the efficiency of epithelial closure over a uniform protein coating is affected by the geometry of the wound^25^. We next considered the role of the geometry of the FN gradient in the gap closure mechanism. We designed a linear FN gradient composed of three successive zones of different FN densities that spread over a total distance of 700 µm (Fig. 4A). Both extremities of the stripe correspond to zones of high FN density with a FN density of 384 ± 10 ng/cm^2^, whereas the central zone corresponds to the zone of low FN density, down to 32 ± 6 ng/cm^2^ (Fig. 4B). As observed on radial FN gradients, MDCK cells attached and spread on zones of high FN density located at both extremities of the adhesive stripe, leading to the formation of a gap located over the low FN density at the center of the pattern (Fig. 4C). The segmentation of catenin-stained tissues showed that MDCK cells increased their spreading areas over the linear FN gradient (Fig. 4D-E), as observed on radial gradients (Fig. 2C). By staining cells with EdU at the final step of the gap closure (Fig. 4F), we observed a higher cell proliferation rate over the lower FN density region of a linear FN gradient (Fig. 4G). This result suggests that cell proliferation is enhanced in freshly closed gaps created over linear FN gradients to regulate the low cell density, as observed for circular gaps. By changing the geometry and the slope (Supplementary Figure S6A–F), we found that the circular FN gradients were characterized with τ = 214 ± 118 min, whereas square and circular step FN gradients were characterized with τ = 202 ± 60 min and τ = 218 min, respectively (Supplementary Fig. S6 G-K). These findings showed that the FN gradient geometry did not statistically affect the gap closure rate. Interestingly, we found that the mean gap closure dynamics (n = 7) on uniform FN coatings with a low density (19 ± 3 ng/cm^2^) was characterized by τ = 176 ± 82 min, whereas the mean gap closure dynamics (n = 7) on uniform FN coatings with a higher FN density (~ 1 µg/cm^2^) was characterized by τ = 106 ± 28 min. (Supplementary Fig. S6 K). Altogether, our findings indicated that the gap closure was statistically delayed when the FN density decreased, suggesting that gap closure dynamics is sensitive to haptotactic gradients.

Taken together, our findings demonstrate that the closure of epithelial gaps in haptotactic conditions is driven by a larger spreading of leader cells, leading to freshly closed gaps with lower cell densities and weaker cell–cell junctions. After the gap closure, a higher proliferation rate over the low FN zone allows to restore the cell density, whereas cell–cell junctions remain weaker in scarred epithelial monolayers (Fig. 4H).

## Discussion

Studying of re-epithelialization, also called gap closure, is crucial for understanding physiological processes, such as wound healing^41^, embryogenesis^42^ and tissue engineering^43^. Different techniques were used to create well-controlled gaps in epithelial tissues, such as scratch assay^44^, laser ablation^45^ or by removing PDMS micropillars^24,46^. Previous reports have revealed the existence of two distinct mechanisms to close a wound in epithelial tissues: cell crawling^24^ and purse string contractions^47^. Purse-string consists in the accumulation of actin and myosin in the first layer of cells located at the frontier of the wound, under the form of a cable connecting neighboring cells from their cell–cell adhesion sites^48,49^. Contractions of this actin cable pushes forward cells from the periphery of the wound to drive epithelial closure. In the crawling mechanism, leader cells extend lamellae^40^ with Arp2/3 mediated branched actin network^50,51^ and close it by crawling^52^. Recently, Vishwakarma and coworkers demonstrated that the emergence of leader cells at the wound frontier is not random, but rather depends on the dynamics of the followers. The number of leader cells being limited by the length up to which they can transmit force, thus depending on both tissue and matrix mechanical properties^53^. Interestingly, these two mechanisms of gap closure are not exclusive^47,54,55^, it was besides shown that purse string and crawling mechanisms coordinate to regulate mechanical work production during wound healing^56^. However the domination of a mechanism over the other seems to depend on the experimental conditions, such as the geometry of the gap to close^25^, the origin of the wound or the presence of injured cells in the wound^24^. Most of the previous works have been performed on uniform coatings of proteins, but in vivo wound healing processes are often related to protein gradients. Interestingly, Vedula^57^ and coworkers demonstrated that the purse string mechanism was involved in the ability of epithelial tissues to close gaps over non-adherent substrates. However, gap closure over non-adherent substrate is only possible for small gaps under a critical diameter of ~ 150 µm, which is moreover cell-type dependent. In addition, the authors showed that geometrical cues modulate the ability of tissues to close over non-adherent substrates^57^. Here, we found that MDCK cells on FN gradients are able to close larger gaps than over non-adherent substrates and that the gap geometry did not affect the closure ability on FN gradients, suggesting the prevalence of a crawling mechanism.

Our findings indicate that leader cells increased their spreading areas to ensure the closure of epithelial gaps on FN gradients. Interestingly, previous reports have shown that cell migration velocity exhibits a biphasic behavior as a function of the ligand density, suggesting that the maximal migration velocity corresponds to intermediate ligand density^7,58^. By varying the concentration of RGD peptides, Abdellatef and coworkers observed that expansion of MDCK tissues increases over time in a substratum-adhesiveness dependent way^59^. These results suggest that cell-substrate adhesions mediated by integrin receptors affect the collective migration of epithelial cells that must extend their spreading to close open gaps over FN gradients, in agreement with our findings.

Focal adhesions at the cell–matrix interface and adherens junctions at the cell–cell adhesions both rely on and share common intermediate proteins and signaling pathways^31,60–62^. Garcia and coworkers^34^ observed a maturation of cell–cell contacts with aging of the tissue. Our findings indicate that the β-catenin fluorescence intensity in mature tissues remains weaker in scarred epithelial monolayers over a FN gradient, in agreement with the weaker cell–cell adhesions found in epithelial cell clusters grown on low RGD coatings^59^. It would be therefore very interesting to study the molecular mechanisms involved into the crosstalk between cadherins and matrix adhesions of epithelial monolayers migrating on protein gradients, even if other factors can intervene in these complex interactions.

Our findings provide a better understanding of collective cell migration on protein gradients, which are reminiscent of haptotactic situations, and suggest a significant influence of the protein density on gap closure, that must be taken into account for further biomaterial engineering.

## Materials and methods

### Design and generation of FN gradients

Square photopattern of 764 × 764 µm was designed with Adobe Illustrator CC 2018 (Adobe Inc.) and consisted in a grayscale radial gradient increasing up to the center. Radial gradients were created between two extreme values of pixel depth set at 85 and 247, respectively. A second photopattern was designed with a rectangular geometry of a 713 × 586 µm and an inverse linear gradient of pixel depth between the same values.

Circular glass coverslips were cleaned by sonication in 70% ethanol solution during 15 min and irradiated under plasma for 5 min (Harrick Plasma, Ithaca, USA). Then a solution of poly-L-lysine at 100 µg/ml (Sigma-Aldrich, Saint-Louis, MO, USA) was incubated on clean coverslips for one hour. Coverslips were washed three times with PBS (Capricorn, Germany) and once with HEPES (Sigma-Aldrich, Saint-Louis, MO, USA) at pH = 8.5 and passivated with a 50 mg/ml solution of PEG coupled with succinimidyl valerate (mPEG-SVA, Laysan Bio Inc., USA) at pH = 8.5 for one hour, followed by 3 PBS rinses. Coverslips were then transferred to the microscope stage and covered with a drop of photosensitive reagent PLPP (Alvéole, France).

PLPP photoreagent was then degraded under UV illumination with a PRIMO^20,21,63^ photopatterning system (Alvéole, France) at a power of 1000 mJ/mm^2^. The coverslip was then incubated for 5 min with a 25 µg/ml^1^ solution of fibronectin from human plasma (Merck Millipore, Germany) and rinsed with sterile PBS (Capricorn, Germany) before use. Photopatterning was controlled by incubating the sample in a solution of rhodamine-labelled fibronectin (Cytoskeleton Inc., USA, ref FNR-01B) to allow its visualization in epifluorescence microscopy.

In order to produce gaps over uniform coatings of FN, we molded circular 1:10 Sylgard 184 (Dow Corning) PDMS stencils^24,25,64^ of 200 μm in diameter from a microstructured silicon wafer, and treated it with Pluronics F127 to avoid protein and cell adhesion at the surface of the PDMS stencil. PDMS stencils were deposited on glass coverslips coated with 200 µl of a 25 µg/ml FN solution for uniform coating with high density, or 200 µl of a 750 ng/ml FN solution for uniform coating with low density, and MDCK cells were plated on the PDMS surface. We let the cells spread and proliferate for 2 days to obtain a confluent epithelial monolayer around the stencils, which was then gently removed to create circular gaps.

### Characterization of the fibronectin gradients

We used the quantification method introduced by Hornung et al.^23^ to obtain a calibration curve of the FN density. We measured the fluorescence intensity of rhodamine-FN solutions (50 ng/ml, 300 ng/ml, 1 µg/ml, 3 µg/ml and 7,5 µg/ml) filled in PDMS microfluidic channels of 200 µm wide and 25 µm thick. The internal side of the microchannels was pretreated with 1% Pluronic F127 solution (BASF) in order to prevent protein adsorption on the internal surface of the channels. Microchannels were filled with each rhodamine-FN solution (Supplementary Fig. S1A) and the fluorescence intensity was measured in similar conditions than those used for imaging rhodamine-FN gradients. After perfusing each rhodamine-FN solution, microchannels were rinsed abundantly with milliQ water and the residual fluorescent intensity corresponding to the adsorbed proteins (residual fluorescence) was substracted to the corresponding fluorescence intensity measurements.

The measured fluorescence signal is due to the total number of molecules inside the microchannel. Using a molecular weight of 250 kDa for Rhodamine-fibronectin (Cytoskeleton Inc., USA, ref FNR-01B), we obtain that 1 µg/ml of Rhodamine -fibronectin corresponds to 2.4 molecules/µm^3^. This volumic concentration can be then assumed as a surface concentration due the microchannel geometry, which is characterized by wide (200 µm) to height (25 µm) aspect ratio of 8.

The corrected fluorescence intensities obtained for 50 ng/ml, 300 ng/ml, 1 µg/ml, 3 µg/ml and 7.5 µg/ml Rhodamine-fibronectin solutions were converted into surface densities to obtain a calibration curve (Supplementary Fig. S1B). We used this calibration curve to estimate the surface density of the rhodamine-FN gradients (according to the blue arrow in Supplementary Fig. S1C). As shown in Supplementary Figure S1D, circular FN gradients ranged from 384 ± 10 ng/cm^2^ at the periphery to 32 ± 6 ng/cm^2^ towards the center, corresponding to the zone of lower FN density (Supplementary Fig. S1D).

Using the same calibration method, we estimated the FN surface densities of patterns with square and circular FN steps, which were characterized by similar mean gray values. We determined that the FN density ranged from 340 ± 14 ng/cm^2^ for the periphery down to 22 ± 7 ng/cm^2^ for the zone of lower FN density at the center of the patterns. In the same way, by comparing the mean gray value to the calibration curve, we determined a surface density of 19 ± 3 ng/cm^2^ for uniform FN coatings with low FN density (Supplementary Fig. S1E).

### Cell culture, seeding and immunostaining

Madin-Darby Canine Kidney cells (MDCK, ECACC, Sigma-Aldrich) were used between passages 36 and 50 ^51^. MDCK cells were grown in a high glucose DMEM medium supplemented with glutamine, 1% of antibiotics/antimycotics and 10% of fetal bovine serum (Capricorn, Germany). Cells were seeded on FN patterns at a concentration of 80,000 cells/cm^2^ and cultured in an incubator at 37 °C and 5% of CO_2_. MDCK cells were fixed with a 4% solution of paraformaldehyde (Electron Microscopy Sciences, Hatfield, PA) and 0.05% Triton X-100 (Sigma) in PBS (Capricorn, Germany) for 15 min at 37 °C and washed three times in PBS. Cells were fixed between 0 to 3 h after closure for “freshly closed tissues” and 36 h after closure for “matured tissues”. Cells were labelled by a first incubation for 45 min at 37 °C with 4,6-diamidino-2-phenylindole dihydrochloride (Invitrogen, Thermofischer Scientific, Waltham, MA, USA) Alexa Fluor 488 Phalloidin (Molecular Probes, Invitrogen) and 1/100 mouse monoclonal anti beta-catenin (clone E-5, Santa Cruz Biotechnology SC-7963). After 3 rinses with PBS, they were incubated for 45 min at 37 °C with a goat anti-mouse antibody labelled with tetramethylrhodamine 1/100 (Invitrogen, Thermofischer Scientific, Waltham, MA, USA). Slides were mounted in Slow Fade Gold Antifade (Invitrogen, Thermofischer Scientific, Waltham, MA, USA).

### Proliferation measurement and inhibition

Proliferation of MDCK cells was assessed using the Click-iT EdU Alexa 647 kit (Invitrogen, Thermofischer Scientific, Waltham, MA, USA). Briefly, cells were incubated with a 10 µM solution of EdU in complete medium for 45 min. Then, cells were rinsed with PBS, fixed for 10 min with a 4% PFA solution and permeabilized for 20 min with a 0.05% solution of Triton X-100. Cells were blocked with 3% BSA and incubated for 30 min in the dark with a reaction cocktail composed of Click-iT reaction buffer, CuSO_4_, AlexaFluor azide and reaction buffer additive. Cells were rinsed and labelled with Hoechst 33,342 (Sigma-Aldrich, Saint-Louis, MO, USA) before mounting in Slow Fade Gold Antifade.

In order to block cell proliferation, we treated cells with a solution of mitomycin C (Sigma Aldrich, Saint-Louis, MO, USA). First, a 0.5 mg.ml^-1^ stock solution of mitomycin C was prepared and stored at 4 °C for a maximum of ten days. Cells were incubated in mitomycin C solution at 5 µg/ml and incubated for 1 h and then rinsed with PBS.

For the quantification of cell proliferation during gap closure, 3 different regions with similar areas were taken into account: (i) the internal border that corresponds to the first rows of cells located at the moving front, (ii) the intermediate zone of the pattern that corresponds to cells located between border of the closing gap and the external side of the pattern and (iii) the external border of the tissues at the border with the non-adhesive coating of the substrate. To quantify cell proliferation, MDCK cells in these three zones were labelled with DAPI^65^ (Sigma-Aldrich) and in Edu-Alexa 647 (Invitrogen, Thermofischer scientific, Walltham, MA, USA).

### Image acquisition and time-lapse recording

Images of immunostained tissues were taken with a Nikon Eclipse Ti-E motorized inverted microscope equipped with × 10 Plan Apo, × 40 Plan Apo (NA 1.45, oil immersion), × 60 Plan Apo (NA 1.45, oil immersion) and × 100 Plan Apo (NA 1.45, oil immersion) objectives and recorded with a Roper QuantEM:512SC EMCCD camera (Photometrics, Tucson, AZ) using NIS Elements Advanced Research 4.0 software (Nikon). MDCK displacements during gap closure were recorded via time-lapse microscopy under the same conditions. During the acquisition, cells were kept in optimal conditions thanks to an incubator placed on the microscope stage, with temperature and CO_2_ controller. Images were recorded every 2 min to follow the gap closure dynamics. Tracking of the cells trajectories was performed with NIS Elements Advanced Research 4.0 software (Nikon, Japan) and analyzed with GraphPad Prism (San Diego, CA, USA).

### Image analysis

The contrast of β-catenin between cell–cell-junctions and the cytoplasm was measured manually with ImageJ from the intensity signal of β-catenin at cell junctions and in the cytoplasm. The ratio of fluorescence between cell junctions and the cytoplasm was calculated for each cell and at least 30 cells were measured for each condition. The contrast of β-catenin intensity was measured for cells inside ROIs corresponding to the low (from 118 ± 8 down to 32 ± 6 ng/cm^2^) and high (FN density from 384 ± 10 down to 118 ± 8 ng/cm^2^) fibronectin density regions of the pattern (Supplementary Fig. S5).

The cellular area was quantified by using the EpiTools software^29^ on β-catenin immunostaining images. First, images were segmented and skeletonized with Matlab 2014a. Then, EpiTools and the bioimaging platform Icy were used to create a map of cellular areas and to export the spreading area corresponding to each cell located inside the pattern (Supplementary Fig. S7). A circular ROI of 350 µm in diameter, centered at the center of the adhesive pattern was used in order to calculate areas in the low (from 118 ± 8 down to 32 ± 6 ng/cm^2^) or high (FN density from 384 ± 10 down to 118 ± 8 ng/cm^2^) fibronectin density areas. In all measurements, the first layer of cells at the border was excluded to eliminate segmentation errors.

### Statistical analysis

Differences in means between groups were evaluated by 2-tailed Student's t-tests performed in GraphPad Prism. For multiple comparisons the differences were determined by using an analysis of variance (ANOVA). *p ≤ 0 0.05, **p ≤ 0 0.01 and ***p ≤ 0 0.001. Unless otherwise stated, all data are presented as mean ± standard deviation (S.D.).

## Supplementary Information

Supplementary Information

Supplementary Video 1

Supplementary Video 2

Supplementary Video 3

Supplementary Video 4
