# Supplementary material for: Collective migration during a gap closure in a two-dimensional haptotactic model
Source: Sci Rep. 2021 Mar 12;11:5811. doi: 10.1038/s41598-021-84998-w (PMC7954790; doi:10.1038/s41598-021-84998-w)
Supplement: Supplementary file 1 — Supplementary Information [file 41598_2021_84998_MOESM1_ESM.docx]

**- Supplementary Information -**

**Collective migration during a gap closure in**

**a two-dimensional haptotactic model**

Marie Versaevel^1^, Laura Alaimo^1^, Valentine Seveau^2^, Marine Luciano^1^, Danahe Mohammed^1^, Céline Bruyère^1^, Eléonore Vercruysse^1^, Olivier Théodoly^2^ and Sylvain Gabriele^1*^

^1^ Mechanobiology & Soft Matter Group, Interfaces and Complex Fluids Laboratory, Research Institute for Biosciences, CIRMAP, University of Mons, 20 Place du Parc B-7000 Mons, Belgium.

^2^ Adhesion and Inflammation Laboratory, INSERM U1067 CNRS UMR 7333, 163 avenue de Luminy - Case 937, 13288 Marseille Cedex 09 France

* Correspondence and requests should be addressed to S.G. sylvain.gabriele@umons.ac.be

**
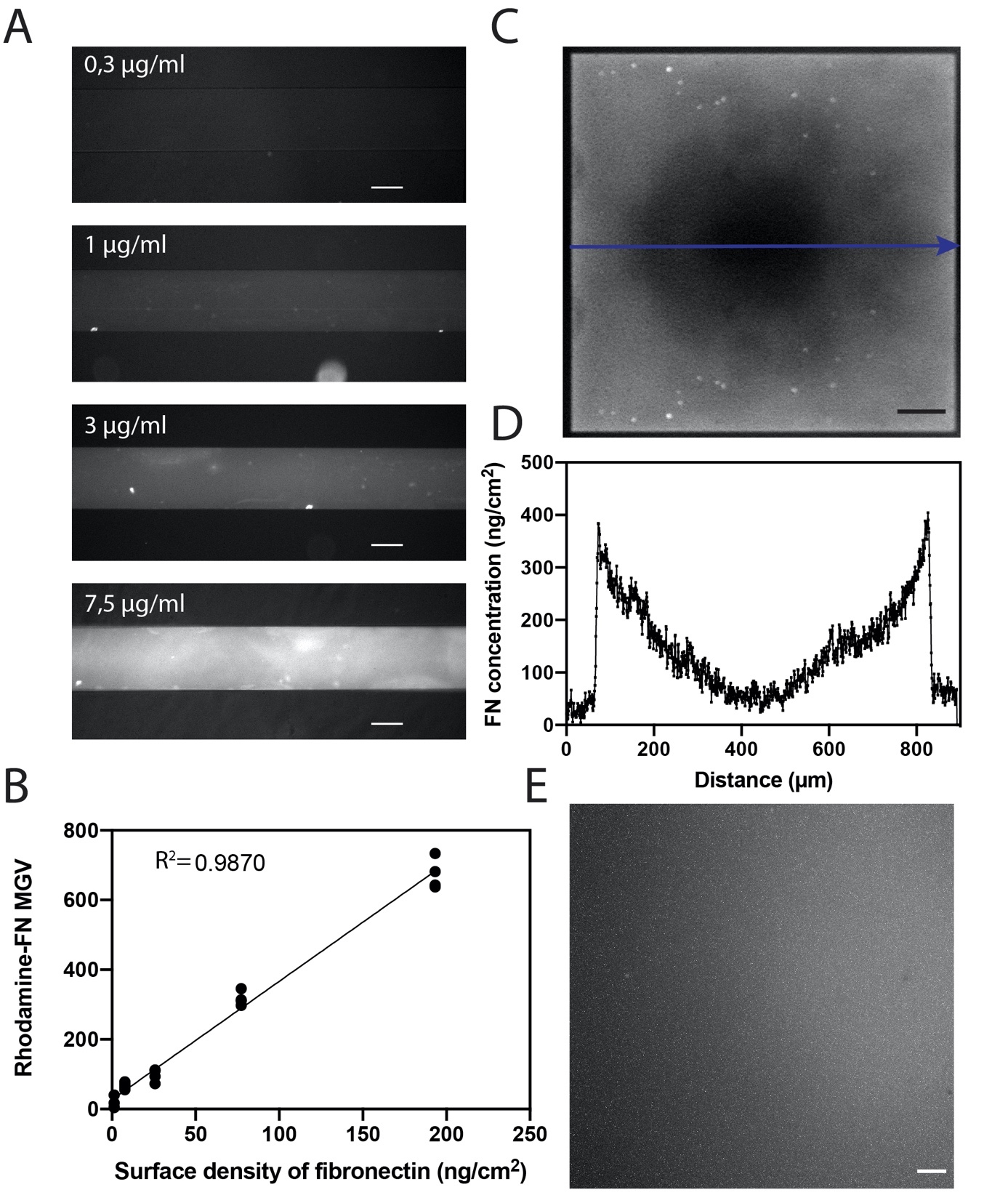
**

**Supplementary Figure S1 – Quantification of the surface concentration of fibronectin on the substrates from microfluidics experiments.** (A) Images obtained in epifluorescence mode of the Rhodamine-fibronectin solutions of various concentrations filled in thin microfluidic channels (scale bars are 100 µm). (B) Calibration curve of the surface concentration of rhodamine-FN as a function of the rhodamine-FN fluorescence signal (mean gray value) obtained from the imaging of the microfluidic channels filled with solutions of various rhodamine-FN concentrations. (C) Epifluorescent image of a FN gradient labelled with rhodamine-FN. Scale bar is 100 µm. (D) FN concentration in the pattern with the gradient of adhesiveness, measured according to the blue arrow in C. (E) Image obtained in epifluorescence mode of the rhodamine-FN solution (750 ng/ml) incubated on the glass coverslips for control stencil experiments with low FN density. The scale bar corresponds to 100 µm.


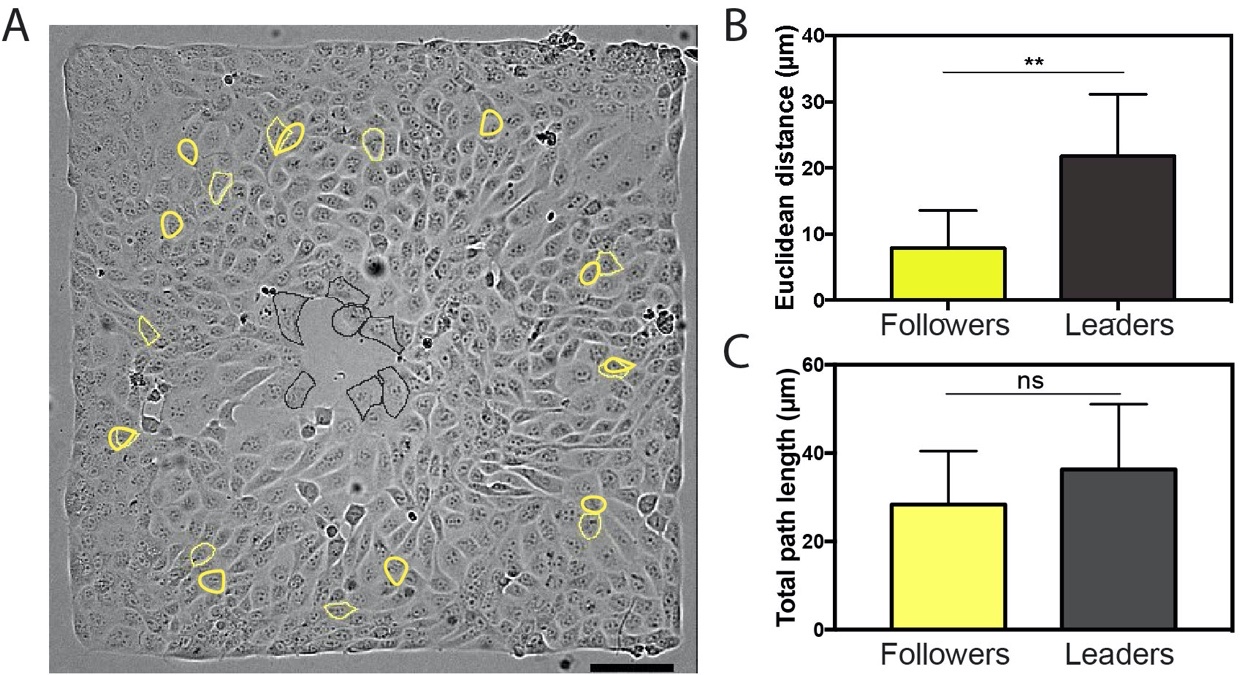


**Supplementary Figure S2 – The persistent motility of leader cells helps closing the gap.** (A) DIC time-lapse of a tissue closing over a circular FN gradient. Leader cells (n=7) tracked for the 100 last minutes of the closing process were highlighted in black. Follower cells (n=10) tracked were highlighted in yellow. The scale bar represents 100 µm. (B) Euclidean distance separating the initial position of cells at the beginning of the tracking and their final position at the end of the closure 100 min later, for leader cells (in yellow, n=7) and follower cells (in black, n=10). (C) Total path length crossed by leader cells (in yellow, n=7) and follower cells (in black, n=10) during the 100 last minutes of the closure process over a circular FN gradient.


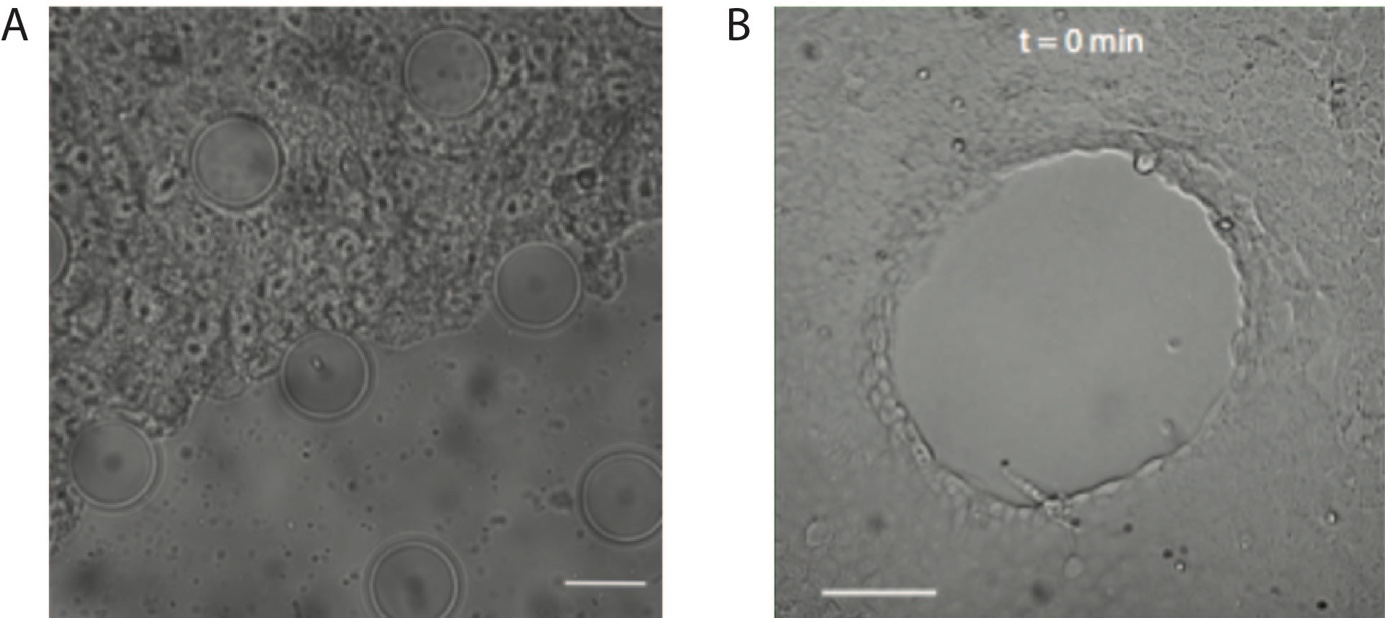


**Supplementary Figure S3 – Control gap closure experiments on uniform a FN coating were obtained from gaps created by a 200 µm diameter PDMS stencil.** DIC images of circular gaps formed by growing MDCK cells around a PDMS stencil deposited on a FN-coated substate. Scale bars are (A) 300 µm and (B) 100 µm.


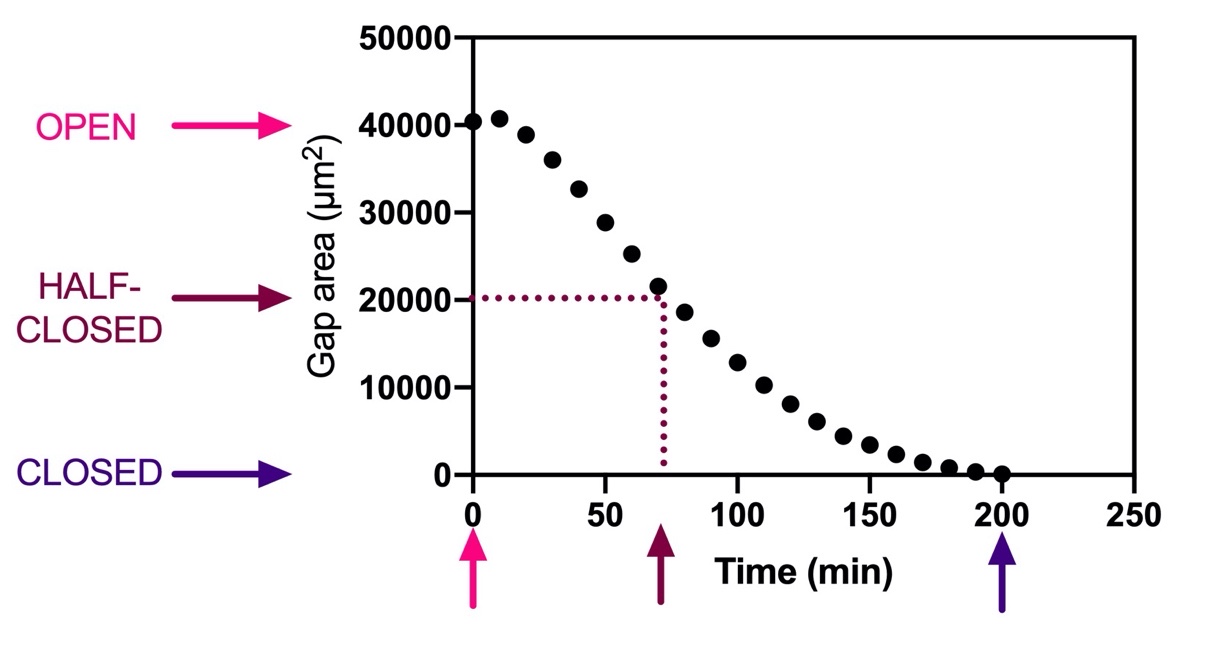


**Supplementary Figure S4 – Temporal evolution of the gap area.** The main stages of the gap closure (open, half-closed and closed) from which cell area were analyzed are indicated with arrows.

**
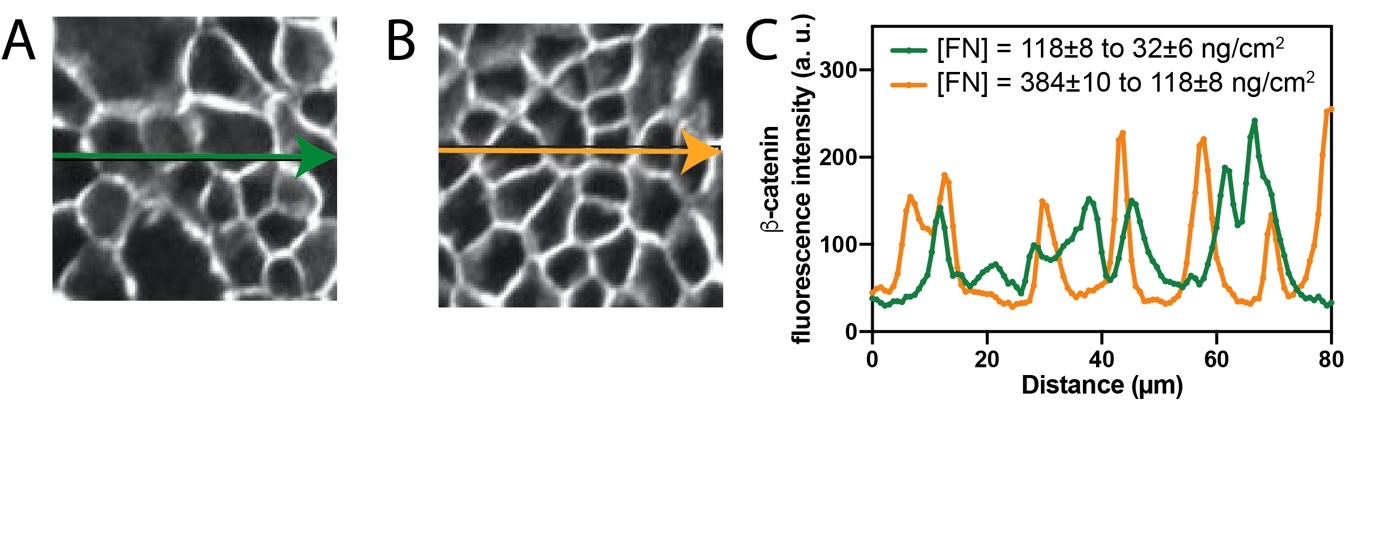
**

**Supplementary Figure S5 – Determination of the cytoplasmic to junctional β-catenin ratio.** β-catenin immunostainings of MDCK cells grown over a FN gradient and matured for 36 h. (A) Plot profile (in green) within the low FN concentration region (from 118 ± 8 down to 32 ± 6 ng/cm^2^) and (B) plot profile (in orange) within the high FN concentration region (from 384 ± 10 down to 118 ± 8 ng/cm^2^). (C) β-catenin fluorescence intensity along green and orange lines.

**
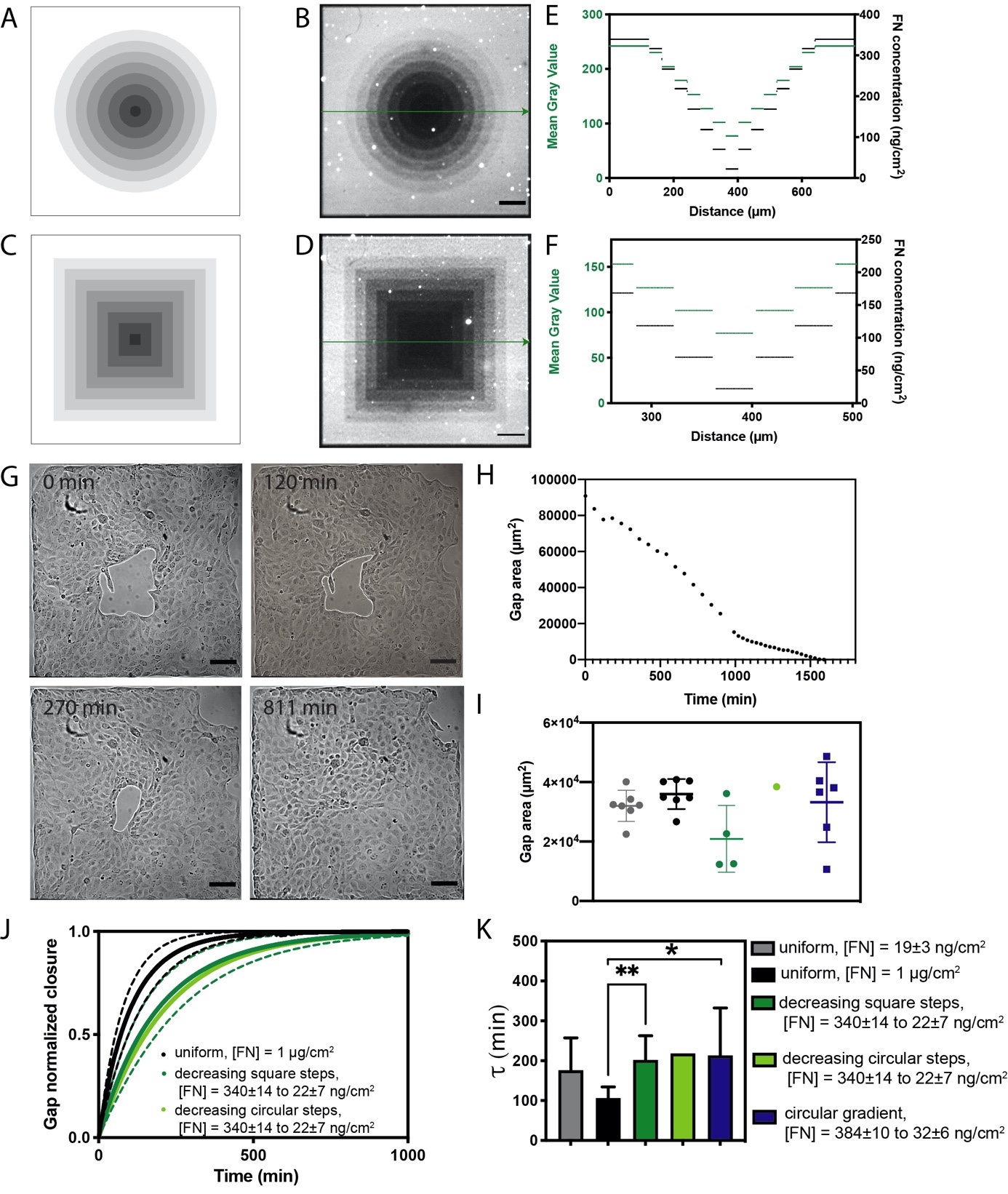
**

**Supplementary Figure S6 – The gap closure dynamics is slowed down by a FN gradient, regardless the gradient geometry.** Photopattern with radial (A) or square (C) step gradient of gray value. (B) and (D) Epifluorescent images of the gradients of rhodamine-labelled FN obtained from the photopatterns in (A) and (B) respectively. Scale bars are 100 µm. (E) Evolution of the mean gray value (left axis) and of the FN surface density (right axis) along the green line in (B) and (D) patterns. (F) Mean gray value and FN density along the green line in (B) and (D) patterns focused on the range of gap sizes. (G) Time-lapse images in DIC mode of a gap closure in a confluent epithelium grown on a pattern with square steps of decreasing FN concentration and obtained from (C). The edge of the closing gap was highlighted with a white line. The scale bar represents 100 µm. (H) Temporal evolution of the gap area during the closure of an epithelium over a FN pattern with square step gradient (from 384±10 to 22±10 ng/cm^2^). (I) Distribution of the gap area in confluent epithelia grown on a uniform coating with low FN density (19$\pm$3 ng/cm^2^, in grey), a uniform coating with high FN density (around 1 µg/cm^2^, in black), a pattern with square steps of decreasing FN density (from 340$\pm14$ ng/cm^2^ to 22$\pm$7 ng/cm^2^, dark green), a pattern with circular steps of decreasing FN density (from 340$\pm14$ ng/cm^2^ to 22$\pm$7 ng/cm^2^ , light green) or a circular FN gradient (from 384$\pm10$ to 32$\pm$6 ng/cm^2^, in blue). (J) Temporal evolution of the mean normalized gap area during the closure on a uniform FN coating (around 1 µg/cm^2^, in black n=7), a square step FN gradient (from 340$\pm14$ ng/cm^2^ to 22$\pm$7 ng/cm^2^, in green n=4) and a radial FN gradient (from 340$\pm14$ ng/cm^2^ to 22$\pm$7 ng/cm^2^, n=1). Full lines represent the mean and dashed lines the S.D. (K) Value of the mean characteristic time for the gap closure in an epithelium grown on a uniform coating with low FN density (19$\pm$3 ng/cm^2^, in grey), a uniform coating with high FN density (around 1 µg/cm2, in black), a pattern with square steps of decreasing FN density (from 340$\pm14$ ng/cm^2^ to 22$\pm$7 ng/cm^2^, dark green), a pattern with circular steps of decreasing FN density (from 340$\pm14$ ng/cm^2^ to 22$\pm$7 ng/cm^2^, light green) or a circular FN gradient (from 384$\pm10$ to 32$\pm$6 ng/cm2, in blue).

**
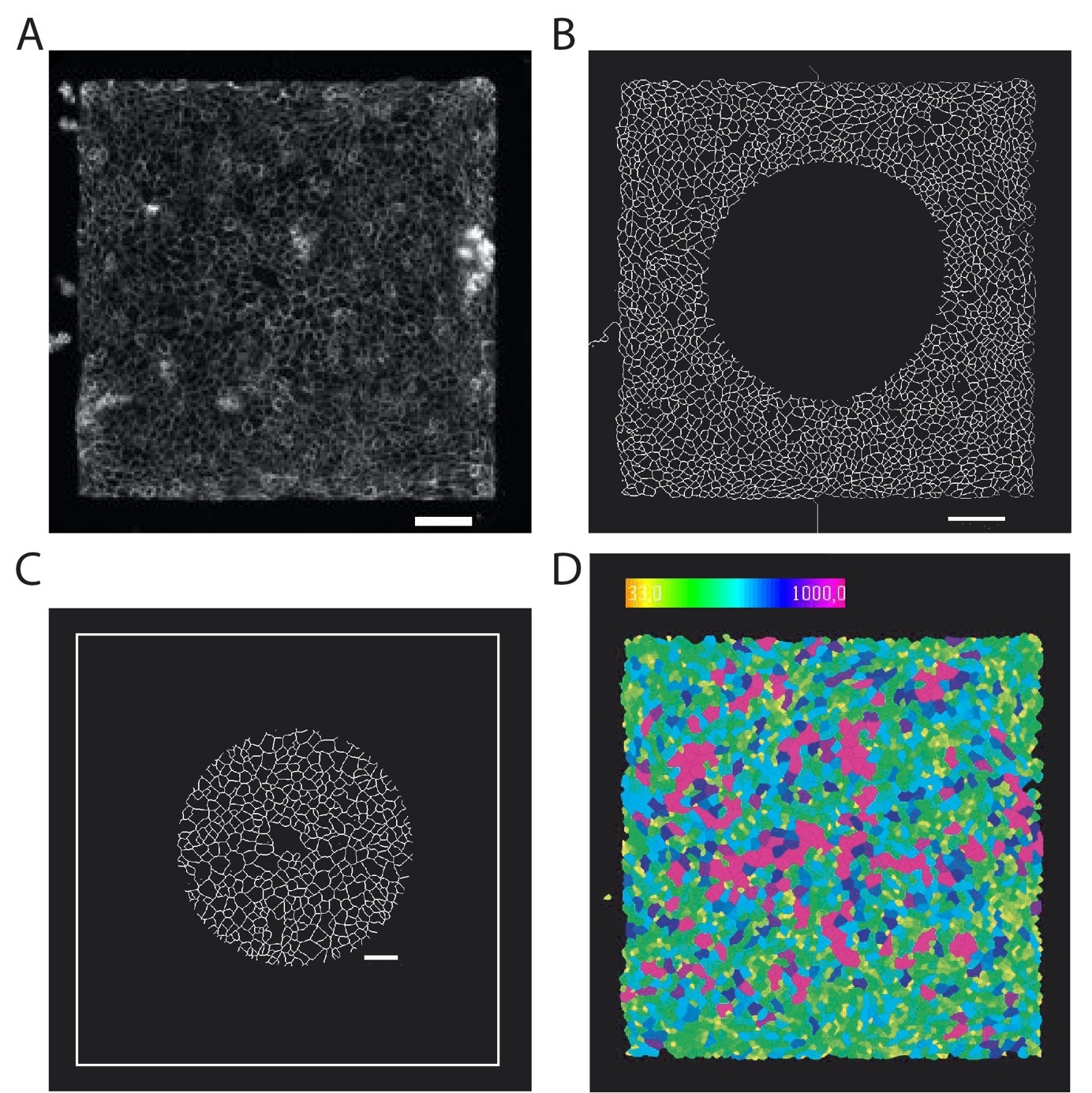
**

**Supplementary Figure S7 – Measurement of the cell area by the EpiTools and Icy softwares.** (A) Epifluorescence image of cellular junctions stained with β-catenin in an epithelial monolayer grown over a radial FN gradient. (B) and (C) Result of the segmentation in zones of (B) high FN density (from 384±10 down to 118±8 ng/cm^2^) and (C) low FN density (from 118±8 down to 32±6 ng/cm^2^). (D) Cell area analysis obtained from the segmented pictures. Smallest cells were labelled in yellow and larger ones were labelled in pink. All scale bars represent 100 µm.

**Supplementary Movie 1 –** Dynamics of closure of an epithelial tissue over a circular FN gradient with a density ranging from 384$\pm10$ down to 32$\pm$6 ng/cm^2^.

**Supplementary Movie 2 –** Dynamics of closure of an epithelial tissue over a uniform FN coating with a density around 1 µg/cm^2^.

**Supplementary Movie 3 –** Dynamics of closure of an epithelial tissue over square step FN gradient with a density ranging from 340$\pm14$ ng/cm^2^ to 22$\pm$7 ng/cm^2^.

**Supplementary Movie 4 –** Dynamics of closure of an epithelial tissue over a uniform FN coating with a density of 19$\pm$3 ng/cm^2^.
